# Gender-Associated Variation in Morphometric Analysis of the Corpus Callosum: A Hospital-Based Cross-Sectional Study Using 3.0 Tesla MRI

**DOI:** 10.7759/cureus.97742

**Published:** 2025-11-25

**Authors:** Arun B Eleyadath, Padamjeet Panchal, Sanjib K Ghosh, Subhash Kumar

**Affiliations:** 1 Department of Anatomy, All India Institute of Medical Sciences Patna, Patna, IND; 2 Department of Anatomy, M.V.J Medical College, Bengaluru, IND; 3 Department of Radiology, All India Institute of Medical Sciences Patna, Patna, IND

**Keywords:** corpus callosum, corpus callosum agenesis, genu, isthmus, morphometry, mri, sexual dimorphism, splenium of corpus callosum

## Abstract

Introduction: The corpus callosum (CC) is a pivotal commissural bundle that facilitates interhemispheric connectivity and contributes to cognitive functions, learning, memory, thinking, visual acuity, executive functions, and behaviour. Precise morphometric dimensions of the CC are imperative for informed surgical interventions, stereotactic approaches, and callosotomy for intractable epilepsy. However, the existing literature lacks consensus on gender- and age-related variations in the CC, with limited representation from Southeast Asia and India. This study aimed to define gender- and age-related variations in CC morphology and establish reliable baseline parameters for clinical and research applications.

Materials and methods: This retrospective, hospital-based cross-sectional study was conducted in the Department of Anatomy. The study included 200 brain magnetic resonance imaging scans, comprising 55% males and 45% females. The brain morphometric parameters such as distance between the anterior frontal pole and the posterior occipital pole (AB), distance between the anterior frontal pole and the anteriormost part of the genu (AC), distance between the anteriormost part of genu to posteriormost part of the splenium (CD) and the distance between the posterior occipital pole and the posteriormost part of the splenium (BD). The CC was divided into five equal subregions along its principal axis. Thickness of the CC was measured at the genu (GL) and at four equidistant body segments (B1, B2, B3, and B4) from proximal to distal. In addition, splenium thickness (SL), callosal height (CC ht), and the splenium index (SI) were also recorded. Measurements were compared across age groups and between genders.

Results: The mean age was 32.93 years, with a similar sex distribution across groups; females showed slightly lower mean ages in older cohorts. Mean CC parameters were comparable between sexes. Males showed slightly higher values in overall AB, AC, and DB. Conversely, females exhibited marginally greater callosal body segmental thicknesses (B1-B4) and CC ht. Sex-wise comparisons using an independent t-test showed no major differences, except for significant variation in splenial length (1-20 years; p = 0.048) and CC ht (21-40 years; p = 0.009). A moderate positive association was observed between CD and AB, as well as between consecutive body segments (B1-B2 and B2-B3). A strong negative correlation was noted between the SI and SL (r = −0.773). Age-related differences were mainly observed in AB, CD, and SL. AB increased significantly from 1-20 to 21-40 years (p = 0.003). CD showed highly significant increases from 1-20 to both 21-40 and 41-60 years (p < 0.001), and a minor difference with 61-80 years (p = 0.01). SL consistently decreased in older groups compared with 1-20 years. SI showed only a marginal change (p = 0.05), indicating overall stability. These findings reflect maturation-related changes predominantly in the anterior and posterior callosal regions.

Conclusion: The study demonstrated measurable gender- and age-related differences in CC, with females showing greater callosal length, thickness, and splenial dimensions, while males exhibited larger brain length and wider interhemispheric distances. Significant correlations among callosal segments and overall brain length further highlighted structural interrelationships. These findings contribute valuable population-specific reference data for anatomical and neuroimaging studies.

## Introduction

For centuries, researchers have explored why men and women differ in cognition and behaviour. While some studies report clear functional differences, such as stronger linguistic skills in women and better mathematical reasoning in men, others remain inconclusive. These variations may partly reflect underlying morphometric differences in the brain. With advances in neuroanatomy and neuroimaging, investigations into gender-related brain morphology have become increasingly precise and rigorous [1]. Among the structures linked to these differences, the corpus callosum (CC) has become a key focus of investigation, serving as the major commissural pathway that enables communication between the two cerebral hemispheres [2].

Composition of the corpus callosum

Anatomically, the CC consists of densely packed myelinated fibres linking distinct cortical regions in an organised yet asymmetric pattern. It is subdivided, from posterior to anterior, into the splenium, body, genu, and rostrum [3]. The genu bends inferiorly and turns posteriorly to continue as the slender rostrum. The rostrum is the anterior part of the corpus callosum that extends toward the anterior commissure. Genu is a prominent anterior convexity interconnecting prefrontal cortices via the forceps minor. The body (trunk) is the central segment linking the frontal, parietal, and portions of the temporal lobes, averaging 6.12 mm in width. The isthmus is the narrowed posterior transition segment of CC situated just anterior to the splenium, adjacent to the fornix. Splenium is a bulbous posterior terminus, forming the forceps major connecting the occipital and temporal cortices [4-6]. The rostrum connects the orbital surfaces of the frontal lobes, while the genu (forceps minor) links the lateral and medial prefrontal cortices. The callosal body is functionally organised: its anterior segment connects premotor and supplementary motor areas; the middle segment links the primary motor cortex with primary and secondary somatosensory cortices; and the posterior segment provides partial interparietal connections. The isthmus primarily carries motor, somatosensory, and auditory fibers. Notably, the central and peripheral regions of the primary visual area lack callosal connections [5]. 

Previous research has suggested sexual dimorphism in splenial dimensions, particularly width [6]. Some studies report that females have a proportionally larger CC cross-sectional area-to-brain ratio; however, others contend that these spurious differences are artefacts arising from brain-size normalisation, given the generally larger cranial volume in males [7]. On average, the CC measures approximately 10 cm in length, half the anteroposterior span of a cerebral hemisphere, and contains an estimated 200-300 million fibres [8].

Topographical relations

The CC forms the roof of the lateral ventricle, lying approximately 4 cm from the frontal pole and 6 cm from the occipital pole. Inferiorly, the fornix descends through the septum pellucidum, while its superior convexity is overlain by the indusium griseum, containing medial and lateral longitudinal striae [9]. The genu constitutes the anterior boundary of the lateral ventricle, while the rostrum continues backwards and downward to join the lamina terminalis. The splenium is anatomically related to the thalamic pulvinar, pineal gland, and midbrain tectum, with the tela choroidea and great cerebral vein of Galen situated inferiorly [10].

Although the CC is indispensable for interhemispheric information transfer, its congenital absence or surgical sectioning does not invariably impair personality or intelligence [11]. However, in congenital anomalies such as complete or partial agenesis of CC, its morphology may be altered. Surgical resections can disrupt the functional integration between the language and perceptual domains. Morphometric variations in the CC have been implicated in the pathophysiology of various conditions, including lipomas [12], glioblastoma multiforme [13], lymphomas [14], demyelinating diseases [15], and vascular lesions [16].

Currently, no universally accepted protocol exists for CC measurement. Magnetic resonance imaging (MRI), especially using high-field 3 Tesla systems, offers a superior modality compared to cadaveric studies, which are confounded by post-mortem shrinkage artefacts. Given that even minute dimensional variations can substantially influence statistical interpretations, precision in imaging and measurement is paramount.

While the global literature on gender differences in CC morphometry is extensive, studies from India, particularly those in the Bihar region, remain scarce. Comparative investigations across populations, such as Sudanese cohorts [17], highlight the need for region-specific normative data. Our retrospective analysis utilises high-resolution 3 Tesla MRI, which provides significantly greater clarity and detail in Digital Imaging and Communications in Medicine (DICOM) datasets than earlier 1.5T studies. Such data have potential applications not only in clinical neurology and neurosurgery but also in genetic counselling, given that agenesis of the CC, one of the most prevalent congenital brain malformations, has an incidence of 0.5-70 per 10,000 individuals [18-20].

CC development begins bidirectionally at approximately six weeks of gestation and is completed between 18 and 20 weeks. Modern imaging techniques can detect agenesis as early as 13 weeks of intrauterine life; however, prognostication remains challenging due to neuroplastic compensatory mechanisms [20]. Given its genetic underpinnings, morphometric analysis of the CC may serve as a surrogate marker for developmental anomalies, neurobehavioral disorders (including autism spectrum conditions), and potential lifelong social and cognitive impairments [21].

Furthermore, the morphometric database generated herein may have implications in forensic medicine, particularly in resolving gender identification disputes. In neurosurgical practice, normative CC parameters can guide interventions such as callosotomies for intractable epilepsy. The integration of high-resolution 3 Tesla MRI, with a slice thickness of merely 1 mm and an enhanced signal-to-noise ratio, affords unparalleled anatomical precision and diagnostic reliability [22]. The inclusion of virtual dissection technology further refines post-processing, enabling exhaustive structural evaluation.

The present study aimed to elucidate gender-associated morphometric variations in the CC, thereby providing baseline anatomical data that may aid in interpreting pathological deviations. The study was conducted to assess gender-related variations in CC dimensions comprehensively, explore interrelationships among its subregions, and examine age-associated differences, thereby establishing a robust normative framework for both clinical and research applications.

## Materials and methods

Study design

This hospital-based, retrospective, observational, analytical, cross-sectional study was conducted in the Department of Anatomy at the Institute. After obtaining Institutional Ethics Committee approval, medical records and brain magnetic resonance imaging (MRI) data of all subjects who underwent MRI examinations at the Department of Radiology between January 2022 and December 2023 were retrospectively analysed.

Patient recruitment and eligibility criteria

The study population consisted of individuals from the Bihar region who underwent MRI studies at the Institute during the specified period. Only MRI scans devoid of any pathologies affecting CC morphology, as confirmed by a radiologist, were retained for analysis.

Inclusion and Exclusion Criteria 

MRI data of the subjects with neurological conditions that do not affect the morphometry of the CC, such as transient ischemic attacks, infections, cerebellar lesions, brainstem lesions, and postoperative follow-up cases not involving the CC, were included.

MRI scans of individuals with established effects on CC morphology were excluded, including congenital brain malformations, encephalomalacia, hydrocephalus, operated cases of intracranial pathology, and a history of cerebrovascular accident.

Sampling and sample size

A consecutive sampling method was utilised, in which all MRI data from individuals who met the predetermined inclusion criteria were systematically included as they became available, until the required sample size was achieved. The sample size was estimated using the G*Power calculator version 3.1.9.4 (Heinrich-Heine-Universität Düsseldorf, Germany) based on the relevant literature [23]. A priori power analysis was performed in G*Power (v3.1.9.4) for a two-tailed independent-samples t-test (means: difference between two independent groups; male vs female) with equal allocation (N₂/N₁ = 1). Using an effect size of Cohen’s d = 0.431 (based on the article) [23], α = 0.03, and desired power (1−β) = 0.80, the required total sample size was N = 198 (99 per group). The corresponding parameters were: noncentrality δ = 3.033, critical t = 2.186, and df = 196; actual power = 0.8009. To accommodate rounding and potential exclusions, the target sample size was set at N = 200.

Data collection and imaging protocol

A total of 200 individuals’ MR images were acquired and evaluated in a mid-sagittal section. The values of the different parameters were assessed in both males and females of varying age groups. MRI scans were performed on a GE Discovery 750 3.0 Tesla scanner (GE Healthcare, Milwaukee, WI, USA) using T1- and T2-weighted protocols with 5 mm slice thickness and 0.5 mm interslice gap. The acquired images were stored in a digital repository and meticulously screened by a radiologist according to predefined inclusion and exclusion criteria.

Following quality screening, the data were reconstructed using Sectra Education Portal ID7 software version 26.1.0.4835 (©2023 Sectra AB, Linköping, Sweden). The Sectra 3D Dissection Table enabled quantitative morphometric assessment in addition to standard 3D visualisation and virtual dissection.

Image analysis

A suitable midsagittal MRI section was selected for measurements. Three observers independently performed each measurement to minimise inter-observer variability, and the mean of their values was recorded.

**Figure 1:**
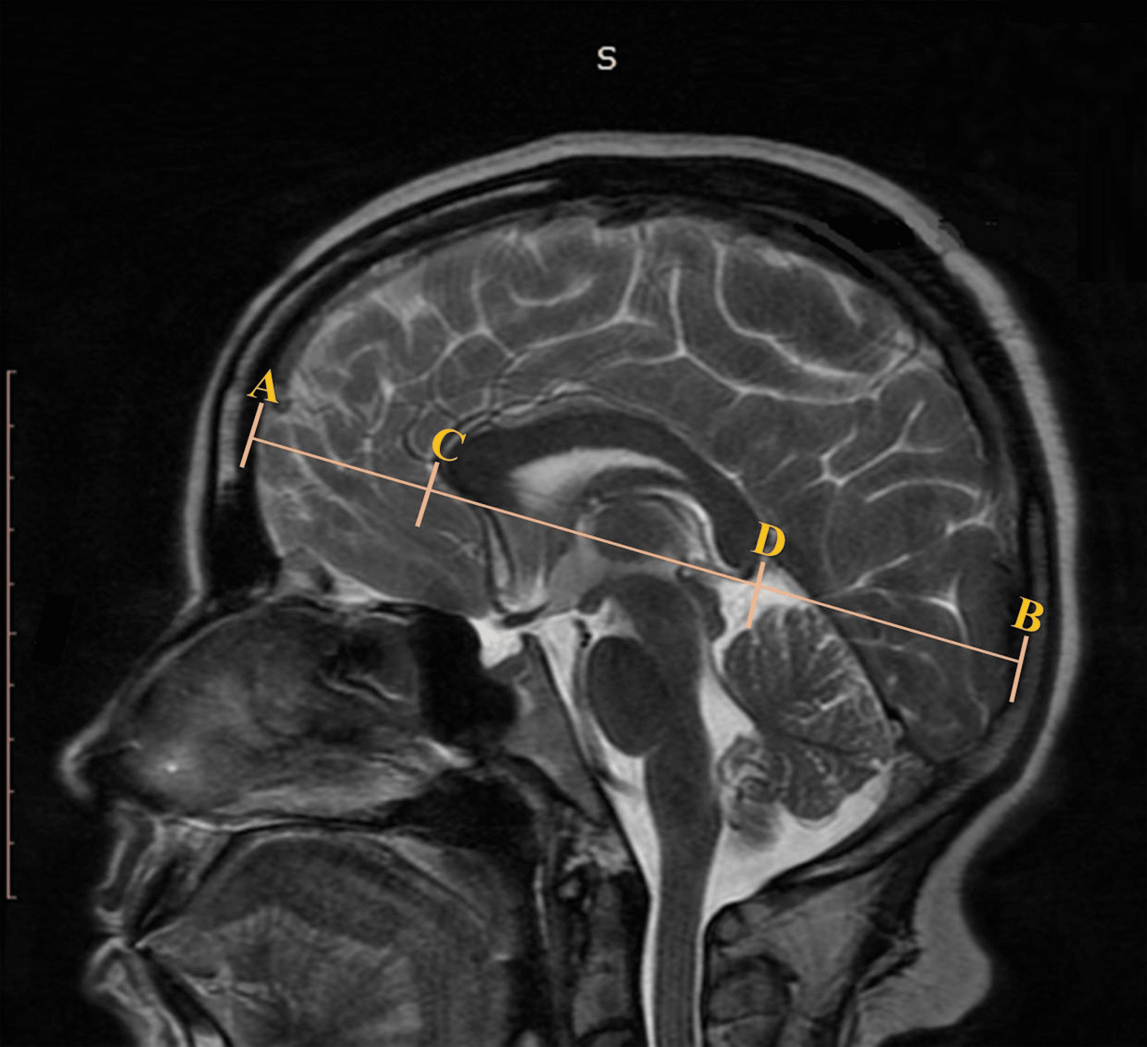
Mid-sagittal T2-weighted MRI of brain shows the main parts of corpus callosum. A- the anterior most point of frontal pole of brain, B- the posterior most point of occipital pole of brain, C - the anterior most part of genu part of corpus callosum, D- the posterior most point of splenium part of corpus callosum, CD- line between the anterior most point of genu (C) to posterior-most point of splenium (D), AB- line between the anterior most point of frontal pole of brain (A) to the posterior most point of an occipital pole of brain (B), AC- line between anterior most point of brain (A) to anterior most point of corpus callosum (C), DB- line between posterior most point of corpus callosum (C) to anterior most point of brain (B), Distance between posterior most point of brain to posterior most point of CC.

Brain morphometric variables included AB, the distance between the anterior frontal pole and the posterior occipital pole of the brain; CD, the distance between the anterior end of the genu and the posterior end of the splenium of CC; AC, the distance between the anterior frontal brain pole and the anterior end of the genu of CC; and BD, the distance between the posterior occipital brain pole and the posterior splenium of CC. The values of these linear distances were measured and noted as shown (Figure 1).

Corpus callosum variables

Morphometric analysis of CC was performed using the method of Weis et al., with modifications to the Witelson Partitioning (WP) scheme [24,25]. The CC was divided into five equal subregions along its principal axis. Thickness of the CC from five different sites, genu (GL), and four equidistant body segments, thickness measurements from proximal to the distal body: Body1 (B1), Body2 (B2), Body3 (B3), and Body4 (B4). Besides this, the splenium length (SL) and CC height (CC Ht) were also measured, and the splenium index (SI) was calculated to assess the shape and proportionality of the splenium. SI is the ratio of CC Ht to SL. 

**Figure 2:**
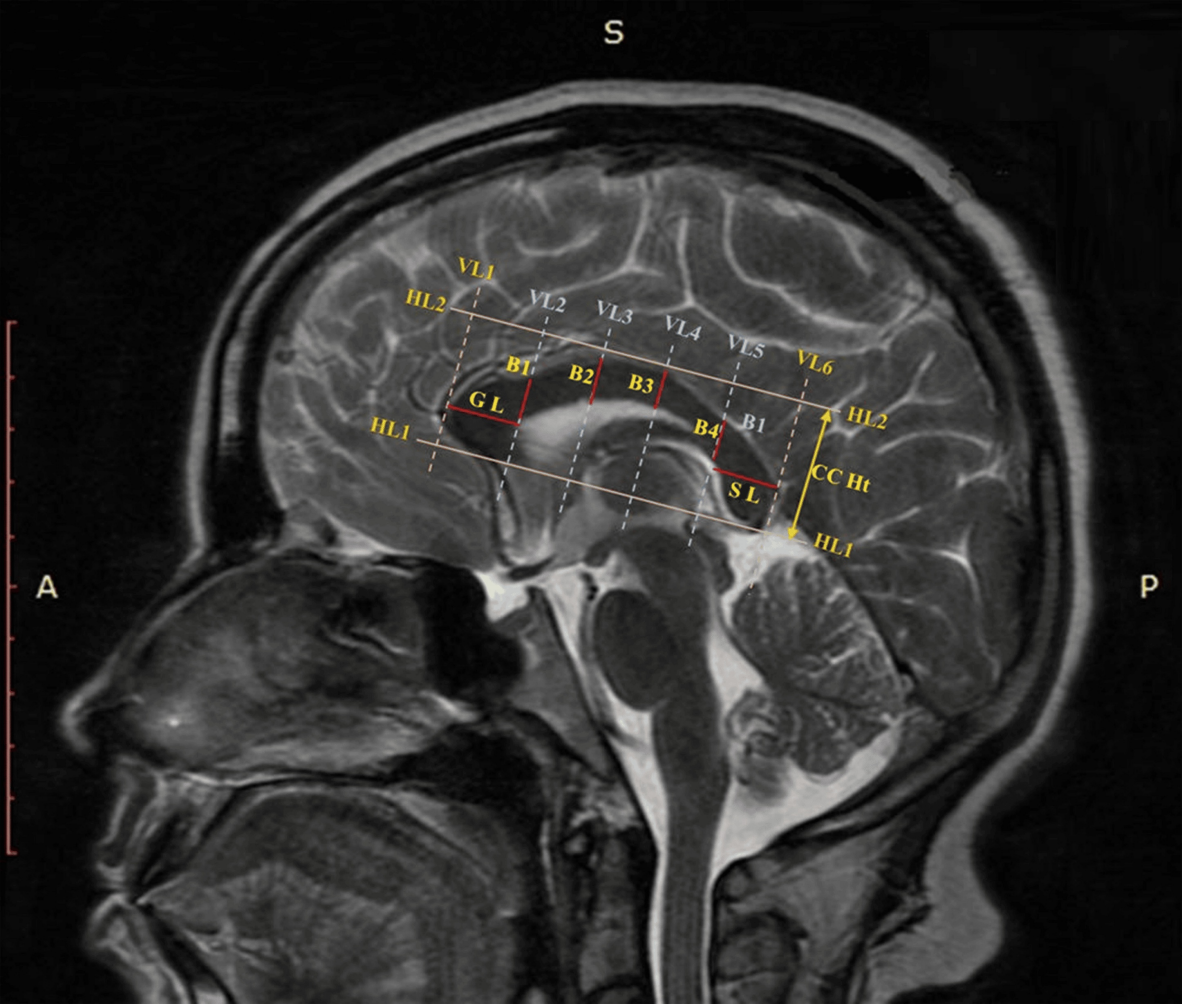
A sagittal T2-weighted MRI of the brain, annotated with multiple linear measurements used in morphometric analysis of the corpus callosum (CC). Reference lines (dashed lines) and the main measurements (solid lines). HL1- Horizontal Line 1 (A tangent connecting the most inferior points of the rostrum and splenium), HL2- Horizontal line 2 (a second reference line following the superior contour of the trunk), VL1- Vertical Line 1 (vertical reference line passing tangentially to the genu and perpendicular to HL1 and HL2),VL2- Vertical Line 6 (vertical reference line passing tangentially to the splenium and perpendicular to HL1 and HL2), Four additional vertical lines VL2- Vertical Line 2, VL3- Vertical Line 3 , VL4- Vertical Line 4, and VL5- Vertical Line 5  which divide the CC into five equal pieces, GL- genu length, B1 to B4- thickness of the first part, second, third and fourth parts of the body of CC, SL – length of the splenium of CC, CC Ht- height of CC (the distance between HL1 and HL2). A modified version of the Witelson Partitioning (WP) scheme [25] was used for taking various measurements.

To establish reproducible morphometric parameters of the corpus callosum, a systematic line-based framework was employed on midsagittal MRI sections. The most inferior points of the rostrum and splenium were first connected by a tangent termed Horizontal Line 1 (HL1). A second tangent along the superior border of the callosal trunk was drawn and designated as Horizontal Line 2 (HL2). The perpendicular distance between horizontal reference lines HL1 and HL2 was defined as CC Ht. A perpendicular line tangent to the genu was marked as Vertical Line 1 (VL1), while another tangent to the splenium was drawn as Vertical Line 6 (VL6). The distance measured between VL1 and VL6 was defined as callosal length (CD). Four additional equidistant vertical lines (VL2-VL5) were constructed between VL1 and VL6, dividing the corpus callosum into five equal segments. The first segment represented the genu, the second to fourth segments denoted the body, and the fifth segment represented the splenium. The genu length was defined as the maximum anterior extension of the genu parallel to HL1. The body thickness parameters (B1 to B4) were obtained from the intersections of the corpus callosum with VL2-VL5. The splenium, which corresponds to the posterior 20% of the corpus callosum, as defined by Weis et al., was measured accordingly (Figure 2) [24].

Measurements of brain and CC morphometric variables were recorded. MRI data collection adhered strictly to ethical guidelines. The acquired MRI morphometric data were categorised into four age groups, each spanning an interval of 20 years, ranging from one to 80 years (i.e., 1-20, 21-40, 41-60, and 61-80 years), to facilitate comparative analysis of age-related variations in corpus callosum morphology.

Statistical analysis

All analyses were conducted on mid-sagittal MRI measurements of CC and brain metrics. Data analysis was performed using SPSS Statistics version 28.0.1 (IBM Corp., Armonk, NY, USA). Formal assessment of normality was employed using the Kolmogorov-Smirnov test for each variable. Homogeneity of variances was examined using Levene’s test for both sex and age categories. Descriptive statistics (mean, standard deviation, range) were computed for each parameter to summarise central tendency and dispersion.

Group comparisons were conducted using parametric or non-parametric tests as appropriate, based on data distribution and variance homogeneity. Sex differences within each age stratum were evaluated using independent-samples t-tests. For age-group effects, one-way ANOVA was applied to each parameter. Where Levene’s test indicated unequal variances, Welch’s ANOVA was used, and degrees of freedom were adjusted accordingly; post-hoc comparisons were then made using the Games-Howell test. Where homogeneity held, the standard (Fisher) ANOVA was followed by Tukey’s HSD for pairwise contrasts. Two-tailed α was set at 0.05 for all hypothesis tests unless otherwise specified.

Inter-relationships among CC and brain measures were examined using Pearson’s product-moment correlation, with significance flagged at conventional thresholds. The correlation matrix was presented to facilitate the interpretation of positive and negative associations among global lengths, segmental thicknesses, and derived indices. 

## Results

Cohort and sex distribution

The mean participant age was 32.93 years, with a comparable male-to-female distribution across groups, and females showed a consistently younger mean age in the older cohorts (Table 1).

**Table 1:** Gender-wise mean age of individuals across different age groups. N - number of individuals, S.D. - standard deviation

AGE Groups (years)	1 TO 20		21 TO 40		41 TO 60		61 TO 80	
GENDER	Females	Males	Females	Males	Females	Males	Females	Males
N	26	46	31	27	23	23	10	14
Mean± S.D.	11.8± 5.45	12.1 ± 5.35	29.3± 5.7	28.8± 6.18	49.6± 5.53	53.6± 4.99	66.1± 3.78	71.0± 6.01

The descriptive statistics of the morphometric parameters of the CC are presented in Table 2.

**Table 2:** Presents mean and standard deviation, and range (minimum–maximum) values of the gender wise morphometric indices of the corpus callosum. Mean CC parameters were comparable between sexes. Males showed slightly higher values in overall AB, AC, and DB. Conversely, females exhibited marginally greater callosal body segmental thicknesses (B1–B4) and callosal height. CD- Length of corpus callosum, AB- The distance between anterior and posterior pole of Brain, AC-The distance between the anterior pole of brain and anterior most point of the corpus callosum, DB- The distance between the posterior pole of brain and posterior most point of the corpus callosum, GL- Thickness of genu, B1 to B4 Thickness of the body of corpus callosum, SL – Thickness of the splenium of corpus callosum, CC Ht- Height of corpus callosum, SI- Splenium index, (mm)- millimeter.

Parameter	Females (Mean)	Females (SD)	Males (Mean)	Males (SD)	Total	Total	
					(Mean)	(SD)	
CD (mm)	70.24	6.165	70.05	7.027	70.13	6.637	
AB (mm)	158.07	9.486	159.96	11.464	159.11	10.636	
GL (mm)	9.83	1.478	9.66	1.759	9.74	1.636	
B1 (mm)	6.04	1.66	5.71	1.44	5.86	1.547	
B2 (mm)	5.3	0.948	4.93	0.975	5.1	0.978	
B3 (mm)	4.73	1.002	4.6	0.979	4.66	0.989	
B4 (mm)	7.71	1.489	7.51	1.845	7.6	1.693	
SL (mm)	10.8	1.778	10.69	1.688	10.74	1.725	
CC Ht (mm)	25.46	2.783	25.1	2.887	25.26	2.839	
SI	2.413	0.4316	2.398	0.4157	2.405	0.4219	
AC (mm)	37.1	3.305	38.33	3.33	37.77	3.367	
DB (mm)	50.33	5.059	52.04	5.652	51.27	5.447	

The Kolmogorov-Smirnov test was applied to evaluate the distributional characteristics of all CC morphometric parameters. All corresponding p-values exceeded 0.05, indicating that none of the parameters deviated significantly from normality (Table 3).

**Table 3:** Showing Kolmogorov-Smirnov test for normality for various parameters. Level of significance p<0.05, KS- Kolmogorov-Smirnov,  D- Maximum Deviation, CD- Length of corpus callosum, AB- The distance between anterior and posterior pole of brain, AC- The distance between the anterior pole of brain and anterior most point of the corpus callosum, DB- The distance between the posterior pole of brain and posterior most point of the corpus callosum, GL- Thickness of genu, B1 to B4 Thickness of the body of corpus callosum, SL – Thickness of the splenium of corpus callosum, CC Ht- Height of corpus callosum, SI- Splenium index.

Parameters	KS (D) value	p value	Level of significance	Normality
AB	0.04535	0.7879	not differ significantly	Normal distribution
CD	0.05499	0.56195	not differ significantly	Normal distribution
GL	0.06542	0.34392	not differ significantly	Normal distribution
B1	0.07797	0.16673	not differ significantly	Normal distribution
B2	0.0718	0.2421	not differ significantly	Normal distribution
B3	0.04913	0.70089	not differ significantly	Normal distribution
B4	0.08396	0.11255	not differ significantly	Normal distribution
SL	0.05038	0.67119	not differ significantly	Normal distribution
CC Ht	0.06461	0.35872	not differ significantly	Normal distribution
SI	0.08019	0.14465	not differ significantly	Normal distribution
AC	0.05299	0.60894	not differ significantly	Normal distribution
DB	0.05622	0.53344	not differ significantly	Normal distribution

Independent-samples t-test was performed to compare mean values across genders within each age group. Across all age group datasets, the CC Ht showed significant sex-related variation (p = 0.009, df = 56), and SL reached significance in the largest cohort (p = 0.048, df = 104). AC and AB repeatedly approached significance (p ≈ 0.05) across multiple groups, indicating a subtle anterior dimorphic trend. All other morphometric parameters showed nonsignificant, consistent results, confirming overall morphometric homogeneity between the sexes (Table 4).

**Table 4:** Comparative t-test summary of gender-wise corpus callosum morphometric parameters results across age cohorts (1–20, 21–40, 41–60, and 61–80 years). * denotes significance level when p<0.05, df- degree of freedom, t value- ratio of the mean difference and standard error. AB- The distance between anterior and posterior pole of brain, CD- Length of corpus callosum, AC- The distance between the anterior pole of brain and anterior most point of the corpus callosum, DB- The distance between the posterior pole of brain and posterior most point of the corpus callosum, GL- Thickness of genu, B1 to B4- Thickness of the first part to forth part of the body of corpus callosum, SL– Thickness of the splenium of corpus callosum, CC Ht- Height of corpus callosum, SI- Splenium index. n.s. = not significant; p < 0.05 considered significant.

Parameter	Age group 1 - 20	Age group 21 - 40	Age group 41 - 60	Age group 61 - 80	Overall Significance Trend
	t-value (df = 104)	t-value (df = 56)	t-value (df = 44)	t-value (df = 24)	
CD	−1.741 (p = 0.085)	0.480 (p = 0.633)	−0.880 (p = 0.384)	−1.244 (p = 0.226)	n.s.
AB	−1.652 (p = 0.102)	−0.776 (p = 0.441)	−1.955 (p = 0.057)	−0.957 (p = 0.348)	n.s.
GL	−0.109 (p = 0.914)	0.489 (p = 0.627)	−0.048 (p = 0.962)	0.281 (p = 0.781)	n.s.
B1	1.906 (p = 0.059)	0.466 (p = 0.643)	0.451 (p = 0.654)	0.355 (p = 0.725)	n.s.
B2	1.651 (p = 0.102)	1.607 (p = 0.114)	1.143 (p = 0.259)	0.884 (p = 0.385)	n.s.
B3	0.247 (p = 0.805)	1.134 (p = 0.261)	−0.745 (p = 0.461)	0.923 (p = 0.365)	n.s.
B4	0.906 (p = 0.367)	−0.581 (p = 0.564)	1.138 (p = 0.261)	−0.089 (p = 0.930)	n.s.
SL	−2.002 (p = 0.048)**	0.482 (p = 0.632)	0.030 (p = 0.977)	−0.016 (p = 0.988)	Significant (age group 1-20)
CC Ht	−1.448 (p = 0.151)	2.711 (p = 0.009)**	0.659 (p = 0.513)	−0.569 (p = 0.575)	Significant (age group 21-40)
SI	0.848 (p = 0.398)	1.066 (p = 0.291)	0.576 (p = 0.567)	−0.498 (p = 0.623)	n.s.
AC	−0.959 (p = 0.340)	−1.952 (p = 0.056)	−1.959 (p = 0.057)	−0.657 (p = 0.518)	n.s
DB	−0.864 (p = 0.389)	−1.049 (p = 0.299)	−1.029 (p = 0.309)	−1.646 (p = 0.113)	n.s.

The Correlation Matrix

The relationships among the variables analysed in the study. The correlation analysis revealed several significant associations among the measured parameters. A moderate positive correlation was observed between CD and AB (r = 0.681), suggesting that as CD increases, AB tends to increase correspondingly. Similarly, moderate positive relationships were identified between B1 and B2 (r = 0.661) and between B2 and B3 (r = 0.637), indicating consistent proportional changes across these parameters. In contrast, a strong negative correlation was observed between SI and SL (r = -0.773), indicating that higher SI values are associated with lower SL values. It is important to emphasise that correlation does not imply causation. While these findings highlight statistically significant associations among parameters, they do not establish the directionality or underlying mechanisms of these relationships (Table 5).

**Table 5:** Shows the correlation between different parameters using Pearson correlation coefficient. *denotes statistically significant correlations level when p < 0.05, ** denotes statistically highly significant correlations level when p < 0.001. AB- The distance between anterior and posterior pole of brain, CD- Length of CC, AC- The distance between the anterior pole of brain and anterior most point of the CC, DB- The distance between the posterior pole of brain and posterior most point of the CC, GL- Thickness of genu, B1- Thickness of the first part of the body of CC, B2- Thickness of the second part of the body of CC, B3- Thickness of the third part of the body of CC, B4- Thickness of the fourth part of the body of CC, SL- Thickness of the splenium of CC, CCht- Height of CC, SI- Splenium index. Pearson correlation coefficients approaching ±1 indicate stronger correlations, while values near 0 denote weak or negligible associations. The diagonal entries, running from the top left to the bottom right, are uniformly 1.0, reflecting the correlation of each variable with itself.

Parameters	CD	AB	GL	B1	B2	B3	B4	SL	CCht	SI	AC	DB
CD	—											
AB	0.681**	—										
GL	0.145*	0.18*	—									
B1	0.038	0.109	0.522*	—								
B2	0.148*	0.128	0.488*	0.66**	—							
B3	0.2*	0.139*	0.455*	0.459*	0.637**	—						
B4	0.263*	0.207*	0.267*	0.357*	0.388*	0.4*	—					
SL	0.538*	0.34*	0.357*	0.176*	0.238*	0.348*	0.488*	—				
CCht	0.531*	0.297*	-0.046	0.169*	0.121*	0.299*	0.302*	0.325*	—			
SI	-0.195*	-0.147*	-0.372*	-0.051	-0.158*	-0.17*	-0.265*	-0.773**	0.315*	—		
AC	-0.028	0.447*	0.001	0.044	-0.042	-0.055	-0.065	-0.054	-0.065	-0.014	—	
DB	0.159*	0.67*	0.143*	0.123	0.087	0.061	0.069	0.017	0.007	-0.001	0.318*	—

An analysis of variance (ANOVA) test was conducted to evaluate age-related differences. The Levene’s test revealed significant differences in variance for AB (F(3,196) = 4.72, p = 0.003), CD (F(3,196) = 2.94, p = 0.034), and B2 (F(3,196) = 2.77, p = 0.043). Consequently, Welch’s ANOVA was applied to these variables. For the remaining parameters, Levene’s test was nonsignificant (p > 0.05), indicating homogeneity of variances; thus, the standard Fisher’s ANOVA was employed. 

The ANOVA Test

Significant differences were observed for AB (Welch’s F = 4.50, p = 0.006), CD (Welch’s F = 9.57, p < 0.001), SL ( Fisher’s F = 7.61, p < 0.001), and SI (Fisher’s F = 3.02, p = 0.031), indicating measurable age-related morphometric variation in both anterior and posterior callosal regions. All other parameters demonstrated nonsignificant differences (p > 0.05), confirming overall stability across midcallosal and curvature dimensions (Table 6).

**Table 6:** Presents Levene’s test results assessing homogeneity of variances and the corresponding one-way ANOVA (Welch’s or Fisher’s, as appropriate) for morphometric parameters of the corpus callosum across four age cohorts (1–20, 21–40, 41–60, and 61–80 years). Welch’s ANOVA was applied when the assumption of equal variances was violated (p < 0.05 in Levene’s test), whereas Fisher’s ANOVA was used for parameters with homogeneous variances. *denotes statistically significant correlations level when p < 0.05, ** denotes statistically high significant level when p < 0.001, df- degree of freedom, F- test statistic of Levene's test, # denotes Performed Welch One-Way ANOVA test (due to unequal variance showing df2 in decimal points), † denotes Performed Fisher One-Way ANOVA test, AB- The distance between anterior and posterior pole of brain, CD- Length of corpus callosum, AC- The distance between the anterior pole of brain and anterior most point of the corpus callosum, DB- The distance between the posterior pole of brain and posterior most point of the corpus callosum, GL- Thickness of genu, B1 to B4- Thickness of the first part to forth part of the body of corpus callosum,  SL- Thickness of the splenium of corpus callosum, CC Ht- Height of corpus callosum, SI- Splenium index.

Homogeneity of Variances Test (Levene's)					One-Way ANOVA			
	F	df1	df2	p value	F	df1	df2	p value
AB^#^	4.72	3	196	0.003	4.50	3	78.3	*0.006
CD^#^	2.94	3	196	0.034	9.57	3	80.7	*< 0.001
GL†	0.146	3	196	0.932	1.41	3	196	0.241
B1†	0.724	3	196	0.540	1.68	3	196	0.172
B2^#^	2.77	3	196	0.043	0.724	3	84.7	0.540
B3†	1.66	3	196	0.178	0.552	3	196	0.647
B4†	0.0850	3	196	0.968	0.137	3	196	0.938
SL†	0.607	3	196	0.611	7.61	3	196	**< 0.001
CC Ht†	2.54	3	196	0.058	1.24	3	196	0.301
SI†	0.388	3	196	0.762	3.02	3	196	*0.031
AC†	0.636	3	196	0.592	0.134	3	196	0.940
DB†	0.683	3	196	0.563	1.54	3	196	0.205

Post Hoc Test

In morphometric analyses, these findings indicate that the most pronounced age-related differences occurred in AB, CD, SL, and SI, with younger age groups (1-20 years) consistently exhibiting shorter lengths compared with older cohorts (Table 7).

**Table 7:** Post-hoc comparison of corpus callosum parameters across age groups using Games–Howell* and Tukey’s# tests. Significant age-related differences were observed in AB, CD, and SL, while SI showed only marginal variation. * denotes Games-Howell Post Hoc Test (homogeneity not met and Welch test significant) # denotes Tukey Post Hoc Test, statistically significant level when p < 0.05, AB- The distance between anterior and posterior pole of the brain, CD- Length of CC,  SL- Thickness of the splenium of CC, SI- Splenium index.

Parameters	Age groups (Years)	Outcome variables across age groups	1 TO 20	21 TO 40	41 TO 60	61 TO 80
AB*	1 TO 20	Mean difference	—	-0.641	-0.426	-0.5869
		p-value	—	0.003	0.066	0.291
	21 TO 40	Mean difference		—	0.215	0.0536
		p-value		—	0.554	0.998
	41 TO 60	Mean difference			—	-0.161
		p-value			—	0.957
	61 TO 80	Mean difference				—
		p-value				—
CD*	1 TO 20	Mean difference	—	-0.558	-0.4718	-0.5464
		p-value	—	< .001	< .001	0.01
	21 TO 40	Mean difference		—	0.0862	0.0116
		p-value		—	0.819	1
	41 TO 60	Mean difference			—	-0.0746
		p-value			—	0.962
	61 TO 80	Mean difference				—
		p-value				—
SL#	1 TO 20	Mean difference	—	-0.107	-0.1196	-0.12569
		p-value	—	0.002	< .001	0.008
	21 TO 40	Mean difference		—	-0.0124	-0.01842
		p-value		—	0.981	0.967
	41 TO 60	Mean difference			—	-0.00605
		p-value			—	0.999
	61 TO 80	Mean difference				—
		p-value				—
SI#	1 TO 20	Mean difference	—	0.19	0.18598	0.164
		p-value	—	0.05	0.085	0.34
	21 TO 40	Mean difference		—	-0.00364	-0.0256
		p-value		—	1	0.994
	41 TO 60	Mean difference			—	-0.0219
		p-value			—	0.997
	61 TO 80	Mean difference				—
		p-value				—

Post hoc comparisons using Games-Howell and Tukey’s tests revealed significant age-related differences in specific corpus callosum parameters. AB showed a significant reduction between the 1-20 and 21-40 years groups (p = 0.003), indicating early age-related enlargement followed by stabilisation. CD exhibited highly significant differences between 1-20 and both 21-40 years (p < 0.001) and 41-60 years (p < 0.001), with a smaller but still significant difference from the 61-80 years group (p = 0.01). For posterior dimensions, SL showed consistent significant decreases in older cohorts compared with 1-20 years, particularly between 1-20 vs 21-40 years (p = 0.002) and 1-20 vs 41-60 years (p < 0.001). The SI showed only a marginal difference between the 1-20 and 21-40 age groups (p = 0.05), suggesting minimal morphological change with age. Overall, significant differences were confined to AB, CD, and SL, reflecting maturation-dependent variation in both anterior and posterior callosal regions, whereas SI remained largely stable across age groups.

## Discussion

The study was carried out over a two-year period utilising MRI data sourced from the radiodiagnosis database at the Institute. The study was structured around three primary research objectives relevant to cerebral anatomy. The first objective was to determine whether morphometric dimensions of the CC exhibit gender-related differences, thereby assessing potential sexual dimorphism in its structural characteristics. The second objective focused on examining interrelationships among CC dimensions to identify significant correlations that could provide insights into its structural organisation. The third objective was to evaluate age-related variations in CC dimensions across defined age groups to elucidate developmental and age-dependent changes throughout the human lifespan. Overall, the findings indicate predominantly modest sex effects, clear age-related differences confined largely to the youngest group, and a coherent pattern of inter-parameter correlations that suggests integrated callosal scaling.

A 3T MRI dataset was used to obtain CC measurements, which were analysed using the Sectra Education Portal (ID7) software. Comparative evaluation with previously reported values demonstrated close agreement, with minor variations likely attributable to ethnic differences and improvements in measurement precision. To ensure methodological consistency, comparisons were primarily restricted to studies that employed MRI, given the inherent limitations of postmortem specimens, such as tissue shrinkage and dimensional changes that occur following removal from the cranial cavity.

The study cohort had a mean age of 32.93 ± 20.85 years, reflecting a predominantly younger demographic. Notably, this age profile differs from those reported in comparable studies. In comparison to the present study cohort (mean age: 32.93 ± 20.85 years), divergence is apparent in other Indian studies, such as those by Suganthy et al. [26] and Murali Krishna et al. [27], which reported average ages of 38 ± 14.8 and 69.59 ± 5.59 years, respectively. Studies from Sudan [17,28] and Turkey [23] documented a mean age ranging from 36 to 47 years, respectively. In contrast, an Iranian study [29] reported a considerably higher mean age of 56 years. Within India, Suganthy et al. [26] reported a mean of 38 ± 14.8 years, whereas Murali Krishna et al. [27] observed a markedly older cohort (69.59 ± 5.59 years). In Nigeria, Ajare et al. [30] reported a mean age of 43.57 ± 19.0 years. In the Arabian population, Allouh et al. [8] reported a mean of 32 years, whereas in Japan, Takeda et al. [31] and in India, Patra et al. [32] documented older means of 61.2 ± 17.6 and 55.84 ± 7.4 years, respectively. Similarly, in Turkey, Tuncer et al. [33] investigated a middle-aged cohort with a mean of 46.6 ± 16.36 years. Conclusively, it is pertinent to note that the studies conducted by Junle et al. [34] and Kosar et al. [35] in Japan, as well as by Varalakshmi et al. [36] in India, reported variations in CC parameters across different age groups in their respective study samples.

CC dimensions were compared with values from recent MRI-based studies across diverse populations, including Sudan, Turkey, Nepal, India, and Nigeria, thereby providing an ethnically representative perspective. For context, two postmortem studies were also incorporated, acknowledging methodological distinctions and their potential impact on morphometric outcomes. Notably, most included investigations utilised MRI scanners ranging from 1.5T to 3T, thereby ensuring comparability with the present study, which employed a 3T MRI scanner. This methodological alignment enhances the robustness of cross-study comparisons and supports the reliability of the findings. Variations in the dimensions of the CC parameters are evident across diverse studies, as highlighted herein (Table 8).

**Table 8:** Summary of corpus callosum (CC) morphometric dimensions and findings reported across global MRI and postmortem studies. Across global studies, CC dimensions show notable variability influenced by ethnicity, age, and methodology. MRI-based research from Sudan, Iran, India, Turkey, Japan, and other regions generally reports CC lengths ranging between 68–76 mm, with genu and splenium widths averaging 9–13 mm. Most studies demonstrate age-related elongation of the CC, while thickness parameters either stabilize or decline with age. Gender dimorphism, when present, is modest—typically limited to slightly larger CC lengths or genu widths in males or females depending on the cohort. Variations among studies likely reflect differences in sample age distribution, imaging strength (0.5T–3T), and population morphology, indicating the importance of population-specific reference data for neuroanatomical assessment. CD- Length of corpus callosum, AB- The distance between anterior and posterior pole of brain, AC- The distance between the anterior pole of brain and anterior most point of the corpus callosum, DB- The distance between the posterior pole of brain and posterior most point of the corpus callosum, GL- Thickness of genu, B1 to B4 Thickness of the body of corpus callosum, SL- Thickness of the splenium of corpus callosum, CC Ht- Height of corpus callosum, SI- Splenium index.

Parameters	Badawi et al. [17]	Mustafa et al. [28]	Mohammadi et al. [29]	Allouh et al. [8]	Gnawali et al. [37]	Ajare et al. [30]	Kosar et al. [35]	Takeda et al. [31]	Junle et al. [34]	Tuncer et al. [33]	Arda et al. [23]	Varalakshmi et al. [36]	Patra et al. [32]	Suganthy et al. [26]	Krishna et al. [27]
Ethnicity	Sudan	Sudan	Iranian	Arab	Nepalese	Nigerian	Japanese	Japanese	Chinese	Turkish	Turkish	Indian	Indian	Indian	Indian
year	2020	2017	2006	2020	2020	2023	2012	2003	2008	2016-2017	2018	2023	2019	2003	2020
Study Method	MRI/3T	MRI /1.5T	MRI/1.5T	3T/MRI	MRI/1.5T	MRI/1.5T	MRI/1.5T	MRI/0.5T	MRI/1.5T	MRI/1.5T	MRI/1.5T	Postmortem	Postmortem	MRI/0.5T	MRI/1.5T
Sample size (n)	385	233	100	227	80	200	90	205	286	104	436	50	50	100	420
AGE (years)	36±18.8	36.72±20.77	56±12.6	32.7±8.8	34±6.2	43.57 ± 19.02	NIL	61.2±17.6	NIL	46.60 ± 16.36	47.05±19.82	NIL	55.84 ± 7.74	38±14.8	69.59±5.59
CD (mm)	76.5±3.6	74.33±7.49	73.09±4.4	68.4±4.0	68±4.8	75.58 ± 4.52	63.54 ± 6.36	69.7±4.24	70.74±4.62	69.82±3.99	68±4.96	68.5 ± 4.7	69.6 ± 5.5	71.6±4.7	69.59±5.59
AB (mm)	161.6±7.2	165.03±11.39	158.3±1.0	151.5±10.8	NIL	161.40 ± 6.0	NIL	NIL	NIL	158.34±4.21	NIL	153.2 ± 8.66	154.7 ± 9.4	152.8±8.4	NIL
GL (mm)	11.12±1.54	11.56±2.39	9.84±1.7	8.8±1.7	9.15±1.6	10.88 ± 1.80	11.54 ± 1.91	9.79±1.90	11.68±1.47	9.72±1.44	10.7±1.83	8.3±1.3	13.1 ± 2.2	10.7±1.9	10.47±1.76
B1 (mm)	6.2±0.82	6.21±1.34	6.84±0.6	5.0±1.0	5.82±0.8	5.63 ± 1.32	6.16 ± 1.39	5.54±1.15	6.20±0.90	5.82±3.12	6.25±1.42	4.4±1.4	4.3±0.8	5.5±0.8	5.36±0.98
B2 (mm)									6.33±0.94		5.81±1.11				
B3 (mm)									4.51±0.86		5.14±1.24				
B4 (mm)									6.33±0.76		10.3±1.98				
SL (mm)	12.82±0.62	10.64±2.18	10.32±1.9	13.5±2.4	10.23±1.6	10.92 ± 1.70	10.42 ± 2.36	9.94±1.56	11.53±1.34	11.19±1.6	13.6±0.99	7.33±1.88	11.8±01.7	11.3±1.6	10.03±1.58
CC Ht (mm)	NIL	NIL	28.52±3.7	NIL	NIL	24.63 ± 3.40	17.57 ± 3.03	25.8±2.84	24.59±2.74	NIL	24.7±3.06	26.1 ± 3.7	NIL	NIL	NIL
SI	NIL	NIL	NIL	NIL	NIL	NIL	NIL	NIL	NIL	NIL	1.81±0.069	NIL	NIL	NIL	NIL
AC (mm)	NIL	36.32±4.22	NIL	NIL	NIL	NIL	NIL	NIL	NIL	NIL	NIL	31.0 ± 2.5	33.1 ± 2.9	NIL	NIL
DB (mm)	NIL	55.98±5.42	NIL	NIL	NIL	NIL	NIL	NIL	NIL	NIL	NIL	54.0 ±5.7	56.5 ± 5.4	NIL	NIL
Findings	The genu length of the CC was significantly greater in females than in males.	Gender-related differences were observed, with males exhibiting larger overall brain dimensions. The callosal index increased with age, and both CC and brain dimensions showed positive correlations with advancing age.	Males had longer genu and splenium lengths than females; however, these differences were not statistically significant. In contrast, CC length demonstrated a significant age-related increase.	Sexual dimorphism was evident, and the CC was found to be smaller in children compared to older age groups.	Variations in CC size were observed across both age and sex. A significant positive correlation was found between age and CC length (p = 0.0019).	Both the length and height of the CC demonstrated significant increases with advancing age.	No statistically significant gender differences were observed in CC parameters when analysed across all age groups.	The widths of the rostrum, body and splenium became thinner with age, and the length and maximum height of the CC increased with age.	No sexual dimorphism. With ageing, significant differences are observed in the widths of the anterior one-third, central region, and posterior one-third.	The parameters showed dimorphism across different ethnicities.	No statistically significant differences were found for gender or age.	The positive correlation between the longitudinal dimension of the brain and all other parameters.	Morphometry of CC will provide baseline data for diagnosing and monitoring disease progression.	CC was longer antero-posteriorly in males, and CC length increased with age, whereas the width of the trunk and genu decreased with age in males but not in females.	There was variability in the thickness and anteroposterior length of CC in accordance with age. The anteroposterior length increased with age.

Specifically, the average length of the CC, denoted as CD, is consistently reported around 70.1 ± 6.6 mm. Noteworthy observations include the extended lengths reported by Badawi et al. [17] and Ajare et al. [30], at 76.5 ± 3.6 mm and 75.58 ± 4.5 mm, respectively. Conversely, studies focusing on the Indian and Nepalese populations by Suganthy et al. [26], Ganwali et al. [37], and Murali Krishna et al. [27] align closely with our findings, reporting CD values of 71.6 ± 4.7 mm, 68 ± 4.8 mm, and 69.59 ± 5.59 mm, respectively. A significant contribution to the global understanding of CC morphometry is the extensive study conducted by Arda et al. [23] on the Turkish population from 2016 to 2018, recording a notable length of 68 ± 4.96 mm. It is pertinent to note that this study employed a 1.5T MRI, while our investigation utilised a 3T MRI, ensuring superior resolving power, precision, and accuracy. Notable concordance is observed in CC dimensions reported by Mustafa et al. (74.33 ± 7.49 mm) [28] and Mohammadi et al. (73.09 ± 4.4 mm) [29] for Sudanese and Iranian samples, respectively. Additionally, Allouh et al. [8] documented an average CC length of 68.4 ± 4.0 mm in the Arabian population. Divergent mean CD values were observed in studies of the Japanese population by Takeda et al. [31] (69.7 ± 4.24 mm) and Kosar et al. [35] (63.54 ± 6.36 mm). Two Indian investigations by Varalakshmi et al. and Patra et al. yielded closely matched results of 68.5 ± 4.7 mm and 69.6 ± 5.5 mm, respectively, similar to the Callosal length reported in a Chinese study (70.74 ± 4.62 mm) [32,36,34]. Lastly, the Turkish study population reported by Tuncer et al. showed a comparable CC length of 69.82 ± 3.99 mm [33]. These findings collectively contribute to the comprehensive understanding of CC dimensions across diverse populations.

In the present investigation, AB was established at 159.1 ± 10.6 mm. It is noteworthy that many studies have focused primarily on measuring the CC, omitting an AB value for comparative analysis. Investigations that incorporate brain length as a metric have demonstrated its predictive utility in elucidating alterations in both the callosal dimension and the relative position of the callosum in response to changes in overall brain size. Consistent with our findings, Suganthy et al. [26] and Mohammadi et al. [29], who conducted studies on the Indian and Iranian populations, respectively, reported congruent values of 152.8 ± 8.4 mm and 158.3 ± 1.0 mm. Conversely, studies by Badawi et al. [17] and Ajare et al. [30] reported slightly larger AB dimensions of 161.6 ± 7.2 mm and 161.4 ± 6.0 mm, respectively, suggesting potential ethnic differences. Mustafa et al. [28] reported a comparable brain length of 165.03 ± 11.39 mm in the Sudanese population. Notably, the study by Allouh et al. [8] revealed a shorter brain length of 151.5 ± 10.8 mm in the Arabian population. It is pertinent to note that brain length was not included as a parameter in the studies by Ganwali et al. [37], Murali Krishna et al. [27], Junle et al. [34], Kosar et al. [35], and Takeda et al. [31]. Additionally, postmortem studies in India by Varalakshmi et al. and Patra et al. reported closely aligned CC lengths at 153.2 ± 8.6 mm and 154.7 ± 9.4 mm, respectively [36,32]. These collective observations contribute to a poor understanding of brain length variation and its implications for callosal dimensions across diverse populations.

The thickness of the genu segment of the CC has been a consistent focus in various studies, including our current investigation. In our study, the average genu dimension was reported as 9.74 ± 1.63 mm. While ranking second-to-last among all reported genu thicknesses, the variance is relatively modest. A study by Patra et al. [32] reported a GL of 13.1 ± 2.2 mm. Comparative analyses with studies conducted in India and Nepal by Suganthy et al. [26] (10.7 ± 1.9 mm), Murali Krishna et al. [27] (10.47 ± 1.76 mm), and Ganwali et al. [37] (9.15 ± 1.6 mm) reveal dimensions larger than those observed in our study. Conversely, Mohammadi et al. [29], investigating the Iranian population, reported a nearly identical genu width of 9.84 ± 1.7 mm, which is consistent with our findings. Noteworthy are the widest genu dimensions reported by Badawi et al. [17] and Mustafa et al. [28] in studies on the Sudanese population, measuring 11.12 ± 1.54 mm and 11.56 ± 2.39 mm, respectively. Arda et al. [23], focusing on the Turkish population, documented a slightly smaller genu width of 10.7 ± 1.83 mm. Conversely, the smallest genu width was observed in the study by Allouh et al. [8] on the Arabian population, reporting a dimension of 8.8 ± 1.7 mm. This aligns with the Indian study by Varalakshmi et al. [36], which reported a GL of 8.31 ± 1.3 mm. Notable genu thickness findings from diverse studies include Ajare et al. [30] at 10.88 ± 1.80 mm, Takeda et al. [31] at 9.79 ± 1.90 mm, Kosar et al. [35] at 11.54 ± 1.91 mm, Junle et al. [34] at 11.98 ± 1.47 mm, and Tuncer et al. [33] at 9.72 ± 1.44 mm. 

The prevailing approach in most studies treats the CC body as a single unit. In contrast, our study adopted a more nuanced perspective by segmenting the CC into four distinct parts, enabling analysis of each part's width. A congruent methodology was observed in the study conducted by Arda et al. [23] on the Turkish population and Junle et al. [34] on the Chinese population. The first segment, B1, exhibited an average thickness of 5.86 ± 1.5 mm in the present study. Comparative investigations have reported slightly higher dimensions, notably 6.25 ± 1.42 mm in the Turkish population [23] and 6.20 ± 0.9 mm in the Chinese population [34]. The latter populations, on the whole, demonstrated greater dimensions, suggesting potential allometric variation compared to the Indian context. Moving to the second segment, B2, our study determined an average thickness of 5.1 ± 0.9 mm. In comparison to the Turkish study [23], B2 in our findings appeared marginally thinner, measuring 5.81 ± 1.11 mm, whereas the Chinese study [34] reported a maximum width of 6.33 ± 0.94 mm. The third segment, B3, displayed a mean thickness of 4.66 ± 0.98 mm, which closely aligned with the Turkish study [23] (5.14 ± 1.24 mm), whereas the Chinese study [34] reported a lower value of 4.51 ± 0.86 mm. The fourth segment, B4, was the largest in the Turkish study [23], with a thickness of 10.3 ± 1.98 mm. There was a significant deviation from our findings, where B4 measured 7.6 ± 1.6 cm. However, concurrence was observed with the study by Junle et al. [34], which reported an average B4 value of 6.33 ± 0.76. Regarding callosum thickness, congruence with our observed values was observed across various studies. Noteworthy examples include Badawi et al. [17] (6.2 ± 0.82 mm), Muhammed et al. [38] from Sudan (6.13 ± 0.92 mm), and the Iranian population [29] (6.84 ± 0.6 mm). Indian studies, particularly by Suganthy et al. [26] (5.5 ± 0.8 mm) and Murali Krishna et al. [27] (5.36 ± 0.98 mm), demonstrated close alignment with our study. In contrast, the study on the Arabian population by Allouh et al. [8] reported a body width of 5.0 ± 1.0 mm, just above the least body thickness reported by Varalakshmi et al. [36] in the Indian population at 4.41 ± 1.4 mm. Additional noteworthy findings were reported by Ajare et al. [30], Takeda et al. [31], Kosar et al. [35], Tuncer et al. [34], and Patra et al. [32], being 5.36 ± 1.32 mm, 5.54 ± 1.15 mm, 6.16 ± 1.39 mm, 5.82 ± 3.12 mm, and 0.43 ± 0.08 cm, respectively. These comprehensive insights contribute to the understanding of CC dimensions across diverse populations.

The average length of the splenium of the CC in our study was determined to be approximately 10.7 ± 1.7 mm. Our findings revealed a dimension lower than that reported in other studies, contributing to the historical debate over splenium length in the CC. Our observations are supported by congruent findings from Indian studies, including Murali Krishna et al. [27], who investigated a sample of 420 individuals using a 1.5T MRI and reported an average splenium length of 10.33 ± 1.58 mm. Suganthy et al. [26] reported a similar figure of 11.3 ± 1.6 mm, while a study in Nepal by Ganwali et al. [37] recorded an average splenium length of 10.23 ± 1.6 mm. This trend also extends to the Iranian population [29], where an average splenium length of 10.32 ± 1.9 mm was observed. Conversely, the Turkish population, as studied by Arda et al. [23], reported the longest splenium, with an average length of 13.6 ± 0.99 mm. Parallel studies on the Sudanese population by Badawi et al. [17] (12.82 ± 0.62 mm) and Mustafa et al. (10.64 ± 2.18 mm) [28] reported comparable lengths. Notably, Allouh et al. [8] investigated the Arabian population and reported a larger splenium length of 13.5 ± 2.4 mm. Significant variation was noted in Indian postmortem studies. Varalakshmi et al. [36] reported an SL of 7.33 ± 1.88 mm, whereas Patra et al. [32], consistent with our study, reported an SL of 11.8 ± 1.7 mm. Additional SL measurements from diverse populations were recorded by Ajare et al. [30] at 10.92 ± 1.70 mm, Takeda et al. [31] at 9.94 ± 1.56 mm, Junle et al. [34] at 11.53 ± 1.34 mm, Tuncer et al. [33] at 11.19 ± 1.6 mm, and Kosar et al. [35] at 10.42 ± 2.36 mm. Overall, splenium length in our study aligns closely with values reported across several Asian populations, while remaining shorter than measurements documented in Turkish and Arabian cohorts.

The exclusive examination of callosum height and splenium index is noted solely in the work of Arda et al. [23], wherein callosum height was reported as 24.7 ± 3.06 mm and the splenium index as 1.81 ± 0.069. Our study, which reveals a slightly increased callosum height of 25.2 ± 2.8 mm and a similar trend in the splenium index at 2.40 ± 0.42, contributes to the limited literature on this subject. Remarkably, Mohammadi et al. [29] documented the largest callosal height to date in their examination of the Iranian population, measuring 28.52 ± 3.7 mm. Comparative analyses with other studies, such as Ajare et al. [30] from Nigeria (24.63 ± 3.40 mm) and Junle et al. [34] from China (24.59 ± 2.74 mm), indicated consistency in callosum height. Notably, Japanese studies displayed considerable variability, as seen in the work of Takeda et al. [31]. Reporting a callosum height of 25.8 ± 2.84 mm, and Kosar et al. [35] reported a callosum height of 17.57 ± 3.03 mm. Validation of our findings is further supported by Varalakshmi et al. [36], who, through the use of cadaveric specimens, reported a callosum height measurement (26.1 ± 3.7 mm) consistent with our investigation. Importantly, our study uniquely focused on delineating the distances AC and DB. This meticulous approach facilitated precise localisation of the CC within the cranial cavity, a dimension often overlooked in prior studies. Thus, our findings show a callosum height comparable to most global reports, with only Iranian and select Japanese data presenting notably higher values.

Gender-based variations were observed across several CC parameters in this study. On average, females exhibited a slightly greater callosal distance (CD: 7.04 ± 0.61 cm) compared to males (7.01 ± 0.70 cm), suggesting a subtle tendency toward longer CCs in females. Conversely, males demonstrated a marginally greater brain length (15.99 ± 1.15 cm) relative to females (15.81 ± 0.95 cm). With respect to genu thickness, females showed a slightly greater mean value (0.983 ± 0.147 cm) compared to males (0.966 ± 0.175 cm), suggesting a tendency toward a thicker genu in the female cohort. Similarly, the mean splenium length was marginally greater in females (1.08 ± 0.177 cm) than in males (1.07 ± 0.169 cm), indicating a subtle proclivity toward a longer splenium. In terms of callosal height, females also exhibited a slightly higher average (2.54 ± 0.278 cm) relative to males (2.51 ± 0.288 cm). Collectively, these findings reflect a modest inclination toward larger dimensions in specific CC subregions among females. When gender differences were examined within specific age groups, notable disparities emerged. In the 1-20 year cohort, significant variation in SL was observed between males and females, whereas in the 21-40 year cohort, differences in CC height were evident. By applying t-tests within age strata, the influence of age on callosal dimensions was minimised, thereby reducing the apparent gender dependence seen in direct mean comparisons. These findings underscore the importance of age-adjusted analyses, as many contemporary studies often overlook gender-associated variations in CC parameters. Several previous studies have also reported gender dimorphism in the corpus callosum, findings consistent with those of the present study. A review of diverse investigations highlights the nuanced nature of this phenomenon, revealing variable outcomes across populations and methodologies, and the influence of multiple contributing factors.

The existing literature presents a complex and often inconsistent picture of gender dimorphism in the CC. While some studies report significant gender-based differences, others have found none [7,23,25,29-31,33-36,39-41]. Meta-analyses by Bishop and Wahlsten [39] and Guz et al. [42] concluded that, overall, there are no statistically significant differences between males and females. However, individual studies included in these analyses reported divergent outcomes [33]. MRI-based investigations have also yielded heterogeneous results, with Johnson et al. noting proportionally larger CCs in females, whereas Pasricha et al. documented greater CC length in males [43,44]. Structural variations have been further highlighted by Allen et al. [45], who observed a more rounded splenium in females and a more cylindrical morphology in males [46]. Much of this variability may be attributed to methodological differences, including variations in sample size, imaging resolution, and whether corrections were made for confounding factors such as brain size. Postmortem studies add further complexity, with Poleneni et al. [41] reporting no dimorphism, while Patra et al. and Varalakshmi et al. identified longer CCs in males and a larger splenium in females [32,36]. Additionally, temporal trends have been suggested, with studies by Sullivan et al. [47] and Allouh et al. [8] indicating that interpretations of gender dimorphism have evolved over time (Table 9) [7,48,49].

**Table 9:** Shows gender associated variations in the corpus callosum (CC) morphometry across different studies. Across studies from 1991 to 2023, investigations into CC sexual dimorphism have yielded inconsistent findings. While some MRI-based studies reported larger CC or genu dimensions in males, and a proportionally larger CC or rounded splenium in females, many others found no significant gender-related differences after adjusting for brain size. Variability in sample size, MRI resolution (0.5T–3T), and analytic methods likely contribute to these discrepancies. Overall, evidence suggests that sex-related differences in CC morphology, when present, are subtle and influenced by age, brain volume, and methodological heterogeneity rather than by true structural dimorphism. CD- Length of corpus callosum, AB- The distance between anterior and posterior pole of brain, AC- The distance between the anterior pole of brain and anterior most point of the corpus callosum, DB- The distance between the posterior pole of brain and posterior most point of the corpus callosum, GL- Thickness of genu, B1 to B4 Thickness of the body of corpus callosum, SL- Thickness of the splenium of corpus callosum, CC Ht- Height of corpus callosum, SI- Splenium index, OASIS- Open Access Series of Imaging Studies, MRI- magnetic resonance imaging

Author(s)	Year	Sample Size	Method	MRI Strength	Key Findings
Allen et al. [45]	1991	146	MRI	0.6T	Splenium rounder in females; more cylindrical in males.
Johnson et al. [43]	1996	263	MRI	0.5T	Females had proportionally larger CC relative to cranial volume.
Bishop & Wahlsten [39]	1997	49	Meta-analysis	—	No gender-based differences in CC size or configuration.
Bermudez et al. [49]	2001	137	MRI	Not specified	men had larger absolute CC areas (total, anterior third, posterior midbody), reflecting overall brain size. greater bulbosity in females.
Sullivan et al. [47]	2001	92	MRI	1.5T	Males had larger CC relative to brain size.
Suganthy et al. [26]	2003	100	MRI	0.5T	CC was longer in males.
Takeda et al. [31]	2003	205	MRI	0.5T	No sexual dimorphism reported.
Mohammadi et al. [29]	2006	40	MRI	1.5T	Males had longer genu and splenium lengths than females.
Junle et al. [34]	2008	286	MRI	1.5T	No sexual dimorphism reported.
Ardekani et al. [7]	2012	316	MRI	1.5T	CC area larger in females after correcting for brain size.
Kosar et al. [35]	2012	90	MRI	1.5T	No sexual dimorphism reported.
Shiino et al. [48]	2017	37	MRI (OASIS DB)	Not specified	CC volume greater in females, especially in the genu.
Mustafa et al. [28]	2017	233	MRI	1.5T	Males had larger brain dimensions and callosal index.
Tuncer et al. [33]	2017	104	MRI	1.5T	No sexual dimorphism reported.
Nair et al. [40]	2017	300	MRI	Not specified	No sexual dimorphism reported.
Arda et al. [23]	2018	436	MRI	1.5T	No sexual dimorphism reported.
Pasricha et al. [44]	2019	200	MRI	Not specified	Males had greater CC length, brain length, and splenium thickness.
Patra et al. [32]	2019	50	Postmortem	—	Longer CC in males; larger splenium in females.
Guz et al. [42]	2019	1108	MRI	Not specified	Significant sex-related differences in CC parameters.
Choudhury et al. [46]	2020	37	Cadaveric study	—	No statistically significant gender differences.
Murali Krishna et al. [27]	2020	420	MRI	1.5T	No sexual dimorphism reported.
Ganwali et al. [37]	2020	80	MRI	1.5T	Variation in CC length with age and sex.
Allouh et al. [8]	2020	227	MRI	3T	No sex-related differences in CC area or thickness.
Badawi et al. [17]	2020	385	MRI	3T	Genu length significantly larger in females.
Soysal et al. [50]	2021	301	MRI	1.5T	Females showed faster decline in CC volume with age.
Poleneni et al. [41]	2021	40	Cadaveric	—	No sexual dimorphism reported.
Ajare et al. [30]	2023	200	MRI	1.5T	No sexual dimorphism reported.
Varalakshmi et al. [36]	2023	50	Postmortem	—	No sexual dimorphism reported.

The relationship between brain parameters and CC morphology has been a subject of considerable interest. In the present study, Pearson correlation analysis revealed a moderate positive association between CC length and overall brain length. Similar moderate positive correlations were observed between adjacent callosal segments (B1-B2 and B2-B3). In contrast, a moderate-to-strong negative correlation was identified between splenium index and splenium length.

In the present study, age-related morphometric differences of the CC were evident across several parameters. Both brain length and CC length showed significant mean differences between the 1-20 and 21-40 year groups, a trend that was also observed when comparing the 1-20 year group with the 61-80 year group. Notably, SL, a parameter that has historically been the subject of considerable debate, demonstrated significant differences between 1-20 years and all other age groups (21-40, 41-60, and 61-80 years). 

Previous studies have also documented age-related morphometric differentiation of the CC. Sullivan et al. reported that CC cross-sectional area increases between five and 18 years of age [47], likely due to progressive myelin sheath thickening of preexisting fibres after early childhood [51]. Nair et al. demonstrated a positive association between age and callosal length in 300 MRI scans [40], consistent with findings by Suganthy et al. [26] and Takeda et al. [31]. This increase in CC size has been attributed to age-related environmental and experiential complexity, which enhances synaptic connectivity and extends the anteroposterior callosal membrane [52]. Sullivan et al. [47] similarly observed callosal lengthening with age, whereas Hopper et al. [53] reported a decrease in CD with advancing age. Takeda et al. [31] also documented an age-related increase in CC height, a finding later corroborated by Gupta et al. [54], who noted a rise in female CC height in postmortem specimens. In contrast, body thickness showed no significant age-related changes [51], though genu and body thicknesses increased until mid-adulthood (around 45 years) before declining [48]. Splenial thickness followed a similar trajectory, peaking around 55 years and subsequently decreasing (Table 10) [52].

**Table 10:** Summary of age-related changes across different morphometry parameters across advancing age, reported across previous MRI and anatomical studies. Across studies spanning nearly three decades, age-related changes in corpus callosum (CC) morphology show a complex but consistent pattern of dynamic structural remodeling. Most investigations report an age-linked increase in anteroposterior length and callosal height, particularly during early adulthood, followed by gradual thinning or reduction in thickness with advancing age. The genu and splenium often exhibit early life variations, while the callosal body shows later-life changes. Some studies also highlight gender-specific patterns, such as increased CC height in females. Overall, evidence suggests that CC dimensions expand during youth and early adulthood, stabilize in midlife, and then progressively decline, reflecting both developmental maturation and age-related neuroanatomical adaptation.

Name of author’s	Year of Study	Age-related variations in CC morphometry
Nair et al. [40]	2017	People's CC lengthens antero-posteriorly as they age, yet it becomes thicker until they're 55, then becomes thinner.
Hopper et al. [53]	1994	Decrease in the length of the CC with advancing age
Takeda et al. [31]	2003	As CC ages, its anteroposterior length and height increase.
Arda et al. [23]	2019	a statistically noteworthy inverse relationship between age and the thickness of both the genu and all sections of the body of the CC
Hadidi et al. [55]	2021	CC parameters showed an increased growth in the first two decades of life. After that, the CC remains roughly the same in the 20-40 age group. After that, the parameters declined over the next two decades.
Sullivan et al. [47]	2002	With age, anteroposterior growth of the CC increases.
Genc et al. [56]	2018	Age-related variation was observed in the length and body thickness of the CC.
Pozzilli et al. [57]	1994	In normal males and girls, the absolute area did not decline appreciably with age. However, a decline in the ratio of the rostrum and genu to the splenium indicated age-related alterations in callosum architecture.
Lebel et al. [58]	2010	The genu and splenium showed age-related changes in the early decades, whereas the callosum body showed changes much later in life.
Ota et al. [59]	2006	No significant change in the thickness of CC’s body with advancing age.
Prendergast et al. [60]	2018	CC area, perimeter, and length undergo dynamic alterations, expanding and contracting as individuals age.
Driesen et al. [61]	2013	The corpus callosum undergoes a minor reduction with age.
Gupta et al. [54]	2008	Increase in the height of the female CC
Pasricha et al. [44]	2023	Increase in the CC's length and a reduction in its thickness with age.
Suganthy et al. [26]	2003	CC length stretching occurs as people age, but the CC body's thickness changes little.

Age-related changes in CC morphology have been variably reported across studies. Several investigations indicate that the anteroposterior length of the CC increases with age, suggesting elongation over time [41,48]. Findings on CC thickness are inconsistent: some report initial thickening until midlife, followed by thinning [41]; others report an inverse relationship with age across all regions [23]; and still others report a progressive reduction [43]. Similarly, changes in CD are debated, with some studies observing an increase [31] and others reporting shortening in older age [51]. Mild age-related reductions in CC size have been reported, particularly in anterior regions [62]. Regional variability has also been documented, with the genu and splenium showing earlier changes and the body demonstrating later-life alterations [55,46]. Gender-specific differences have been noted, including increased CC height in females but not males [54]. Some authors further emphasise dynamic alterations in CC area, perimeter, and length across the lifespan [58], while others report stability in body thickness despite advancing age [57]. The compilation of studies on age-related variations in CC morphometry highlights the complex trajectory of structural changes across the lifespan. Alterations in anteroposterior length, thickness, transverse diameter, and regional subcomponents revealed the heterogeneous nature of CC ageing. While some investigations report progressive growth or elongation, others emphasise region-specific or even opposing trends, reflecting expansion and contraction in CC dimensions at different life stages. These dynamic patterns, further influenced by gender differences, illustrate the multifactorial nature of CC remodelling. Collectively, the evidence highlights the role of genetic, environmental, and individual factors in shaping CC morphology and emphasises the need for continued research to clarify the neurobiological basis and cognitive implications of age-related changes in interhemispheric connectivity. 

Methodological considerations

Two methodological choices strengthen the present analysis. First, the use of 3T MRI and digital measurement (Sectra ID7) likely yielded higher spatial precision than older 0.5T-1.5T studies. Second, segmenting the CC body into four parts allowed us to show that not all midcallosal regions behave identically across age groups - a pattern often missed when the CC body is treated as a single entity. At the same time, the need to apply Welch’s ANOVA for some variables indicates that variance heterogeneity is common in age-stratified morphometric datasets, and justifies the use of Games-Howell rather than default Tukey tests in those instances.

Limitations****

The present study has certain limitations that warrant consideration. First, only a limited number of comparable studies could be included, as very few met the specific methodological criteria. Additionally, the raw data from the published studies were not available, which restricted further in-depth analysis. The limitations include its retrospective design and single-centre data. MRI itself is an operator-dependent technique, making it, to some extent, susceptible to detection and measurement errors. Brain MRI findings may be influenced by technical and patient-related factors such as motion artifacts, sequence selection, slice-positioning inaccuracies, and susceptibility effects. These limitations can reduce image clarity and potentially affect the precision of morphometric measurements. Furthermore, the process of uploading MRI scans into SECTRA ID7 software proved time-consuming and challenging, particularly during busy academic hours, potentially affecting workflow efficiency. The age distribution was skewed to younger adults, which may limit direct comparability with studies whose samples cluster in the fifth to seventh decades. Finally, some comparator studies did not report all CC subcomponents (AB, AC, DB), so cross-study contrasts could not always be made on the same set of variables.

Implications

The findings of this study support an advanced understanding of the corpus callosum developmental model in which (i) most linear dimensions reach near-adult values early, (ii) sex-related differences, when present, are small and age-sensitive, and (iii) the CC enlarges as an integrated structure, with shape adapting to length. These results provide normative morphometric data for the Indian population and can serve as a reference in studies of neurodevelopmental, neurodegenerative, and demyelinating disorders where callosal integrity is affected. Future studies using larger, age-balanced MRI cohorts and brain-size-corrected analyses would help disentangle true sex dimorphism from age and cranial-size effects and clarify the clinical relevance of these morphometric patterns.

## Conclusions

The study demonstrates that corpus callosum morphology exhibits measurable variability across gender and age groups in the northern Indian population. Analysis revealed subtle gender-related variations in morphometric features, including differences in overall length, brain dimension, anterior and posterior interhemispheric distances, thickness across multiple body segments, splenium length, corpus callosum height, and splenium index values. Females generally presented a relatively longer corpus callosum, with greater thickness across most segments, a larger splenium, and increased callosal height. The splenium index also appeared slightly higher in females. In contrast, males exhibited comparatively greater overall brain length, along with larger anterior and posterior interhemispheric distances. Age-related assessment revealed variability in splenium length and callosal height across different life stages, with distinct differences observed between younger and middle-aged groups. Positive correlations between callosal length, brain length, and several callosal segments, along with the inverse relationship between splenium index and callosal length, further strongly indicate the structural interdependence within callosal architecture. Overall, these findings provide meaningful insights into population-specific neuroanatomical variation and may serve as a comparative reference for future clinical, anatomical, and imaging studies investigating callosal morphology.
